# Prognostic value of the standardized uptake value for ^18^F-fluorodeoxyglucose in patients with stage IIIB melanoma

**DOI:** 10.1007/s00259-012-2182-0

**Published:** 2012-07-17

**Authors:** E. Bastiaannet, O. S. Hoekstra, J. R. de Jong, A. H. Brouwers, A. J. H. Suurmeijer, H. J. Hoekstra

**Affiliations:** 1 Department of Surgical Oncology, University Medical Centre Groningen, University of Groningen, PO Box 30.001, 9700 RB Groningen, The Netherlands; 2 Nuclear Medicine & PET Research, University Medical Centre, VU Amsterdam, Amsterdam, The Netherlands; 3 Nuclear Medicine and Molecular Imaging, University Medical Centre Groningen, University of Groningen, Groningen, The Netherlands; 4 Department of Pathology, University Medical Centre Groningen, University of Groningen, Groningen, the Netherlands

**Keywords:** Melanoma stage III, Lymph node metastases, FDG PET, SUV

## Abstract

**Purpose:**

FDG PET/CT is an excellent tool to detect melanoma metastases and also allows quantification of FDG uptake using standardized uptake value (SUV). The aim of this study was to prospectively investigate the potential prognostic value of SUV for disease-free survival (DFS) and disease-specific survival (DSS) for patients with stage IIIB melanoma.

**Methods:**

From November 2003 to March 2008, all consecutive patients were included in the present study. Inclusion criteria were: palpable, histology- or cytology-proven lymph node metastases of melanoma, and referred to the University Medical Centre Groningen for FDG PET and CT examination. Patients without distant metastases were evaluated. Multivariable survival analysis was performed to determine whether SUV was associated with DFS and DSS (Cox proportional hazard analysis).

**Results:**

In 80 patients (without distant metastases, 65 %) SUV could be measured. Overall 5-year DFS was 41 % (95% CI 26–56 %) and 24 % (95% CI 12–38 %) in patients with a low and high SUVmean (*p* = 0.02), respectively. Overall 5-year DSS was 48 % (95% CI 31–62 %) and 30 % (95% CI 17–45 %) in patients with a low and high SUVmean (*p* = 0.04), respectively. In the multivariable analysis, SUVmean was associated with DFS (hazard ratio 1.7; *p* = 0.048), but was not associated with DSS (hazard ratio 1.6; *p* = 0.1). The number of positive nodes, extranodal growth and gender were also associated with survival.

**Conclusion:**

FDG uptake in clinically overt nodal melanoma metastases is inversely associated with DFS. Univariate analysis showed an association with DSS. However, after adjustment for potential confounders this association was no longer significant. If these findings are confirmed in larger studies, SUVmean could potentially be used (in addition to the number of positive nodes, tumour size and extranodal growth) as a factor in deciding on adjuvant systemic treatment.

**Electronic supplementary material:**

The online version of this article (doi:10.1007/s00259-012-2182-0) contains supplementary material, which is available to authorized users.

## Introduction

Melanoma is a tumour with one of the most rapidly increasing incidence rates, especially in Caucasian populations. In a decade, its incidence in the Netherlands has risen by 63 % to 21.7/100,000 (European standardized rate) from 13.3/100,000 in 1998 [1]. Standard treatment for patients with palpable lymph node metastases (AJCC stage IIIB) is therapeutic lymph node dissection (TLND). However, these patients are at high risk of developing distant metastases. Established prognostic factors in patients with stage IIIB melanoma are the number and size of metastatic nodes, and the presence of extranodal growth [2, 3]. Identification of additional prognostic factors could lead to more individualized treatment and follow-up schemes.

Whole-body ^18^F-FDG PET is a sensitive screening tool in patients with high-risk melanoma, since melanomas are typically FDG-avid [4]. The added value of FDG PET over CT with respect to diagnostic accuracy and impact on management has been established in several studies [4–10]. Besides improved detection of metastases, a standard clinical PET/CT scan offers the opportunity to quantify the level of glucose metabolism in tumours. Glucose metabolism, as assessed with FDG, is an epiphenomenon of cancer and seems to have prognostic value [11–17]. However, the value of FDG as a prognostic biomarker is not undisputed [18], probably as a consequence of confounding and heterogeneity in both the clinical spectrum and PET technology. We have reported some preliminary data acquired in a retrospective setting suggesting an inverse relationship between tumour FDG uptake in patients with stage III melanoma who receive surgery with curative intent [13]. The present study aimed to prospectively validate these findings taking into account the above-mentioned potential confounders.

## Methods

### Patients

From November 2003 until March 2008, all patients with palpable, histology- or cytology-proven lymph node metastases of melanoma referred to the University Medical Centre Groningen (UMCG) for FDG PET and CT examination were prospectively included in this study. Prior to study entry, all patients were considered candidates for TLND, and they took part in a large prospective multicentre study to assess the diagnostic performance of FDG PET and multislice CT [4]. Inclusion criteria for the present study were: stage III after FDG PET and abdominal/thoracic CT, candidate for TLND with curative intent, and scanned on the same FDG PET scanner with identical patient preparation, acquisition and reconstruction protocols. Patients with diabetes were not included. The study was approved by the UMCG Medical Ethics Committee. Patients were actively followed up until January 2012. Follow-up was uniform in all patients and consisted of clinical evaluation of physical signs and symptoms at standard follow-up visits (every 3 months in the first year, every 4 months in the second year, twice a year in the third to fifth years, and every year in the sixth to tenth years); diagnostic tests (chest radiography, CT or FDG PET) were only indicated if distant metastases were suspected.

### FDG PET and SUV

FDG was synthesized on-site according to the method described by Hamacher et al. using an automated synthesis module [19]. Prior to FDG PET imaging, patients were instructed to fast for at least 6 h and to drink 1 l of water. After intravenous injection of FDG, whole-body imaging was performed in the two-dimensional mode using a Siemens ECAT EXACT HR+ scanner (Siemens/CTI, Knoxville, TN), applying emission scans of 5 min per bed position, starting 90 min after the injection of FDG. Image reconstructions were iterative (ordered subset expectation maximization ) with eight subsets and two iterations. The image matrix size was 128 and the voxel size was 5.0. All scans were corrected for decay, scatter, randoms and attenuation, and normalized. The postreconstruction filter (gaussian postprocessing filter) was 5 mm, yielding an estimated spatial resolution (full-width at half-maximum) of 7 mm.

FDG uptake was calculated as SUV (radioactivity concentration in tissue in becquerels per cubic centimetre/injected dose in becquerels/patient body weight in grams). Three-dimensional volumes of interest were placed semiautomatically around the lymph node metastasis with the highest FDG uptake (visual assessment) using dedicated software (IDL-Viewer, Leuven). The numerator of the SUV was chosen to measure either the mean counts within an isocontour to 70 % of the voxels with the highest counts, or the maximum itself, yielding SUVmean or SUVmax, respectively.

### Data analysis

To explore the distribution of SUVmean and SUVmax a kernel density plot was used, which approximates the probability density of the variable; Kernel density plots have the advantage of being independent of the choice of origin, unlike histograms. The associations between SUVmean/SUVmax or the log-transformation (which gave the best fit) and patient characteristics were assessed using Student’s *t*-test or analysis of variance. Patients were divided into two groups (high and low SUV) based on the median value of the log(SUVmean) and log(SUVmax) for the survival analysis. This dichotomization led to the same patient categorization for SUVmax and SUVmean in 95 % of the patients. Since repeatability of SUVmean is superior to that of SUVmax [20, 21], we show the SUVmean results in the analyses.

Survival was analysed in terms of disease-free survival (DFS) and disease-specific survival (DSS). Differences in survival were assessed using the log-rank test. For DFS an event was recorded for any recurrence; for DSS an event was recorded if the patient died due to melanoma metastases. Multivariable Cox proportional hazard analysis was performed to determine independent prognostic factors. Multivariable models were created with age, gender, location of the lymph nodes defined as cervical region, axilla or groin, number of nodes removed during TLND, number of lymph nodes positive on histopathology after TLND, tumour size in the largest lymph node metastasis determined after TLND by histopathology, extranodal growth defined as metastatic tumour which clearly extended (histologically) through the nodal capsule into the perinodal fatty tissue or tumour involvement in the hilar region with interruption of the smooth outline of the (presumed) capsule, Breslow thickness and ulceration of the primary melanoma, and high or low SUVmean. The proportional hazard assumption was tested for the multivariable models. All *p*-values were considered significant if *p* < 0.05.

## Results

A total of 80 patients were included in the present study. There were no patients lost to follow-up. Table 1 shows the characteristics of this cohort. There were slightly more women: 41 women (51.2 %), 39 men (48.8 %). Their median age was 57.0 years (range 24.7–93.2 years). Lymph node metastases were located in the groin (55.0 %), axilla (32.5 %) or cervical region (12.5 %). The median number of removed nodes was 16 (range 7–48) and the median number of positive nodes was 2 (range 1–19). The tumour size in the lymph node ranged from 0.5 to 7.0 cm with a median of 3.1 cm. In 25 patients (31.2 %) extranodal growth was recorded.

**Table 1:** Patient and tumour characteristics of the 80 patients with palpable, histology- or cytology-proven lymph node metastases of melanoma

Characteristic		Value
Gender, *n* (%)	Men	39 (48.8)
	Women	41 (51.2)
Age (years), *n* (%)	<50	25 (31.2)
	50–65	32 (40.0)
	>65	23 (28.8)
Primary melanoma		
Location, *n* (%)	Upper extremities	9 (11.2)
	Lower extremities	34 (42.5)
	Trunk	29 (36.3)
	Head and neck	6 (7.5)
	Unknown primary	2 (2.5)
Breslow thickness (mm), *n* (%)	≤1.0	10 (12.5)
	1.0–2.0	27 (33.8)
	≥2.0	41 (51.2)
	Unknown primary	2 (2.5)
Ulceration, *n* (%)	No	63 (78.8)
	Yes	15 (18.7)
	Unknown primary	2 (2.5)
Lymph node metastases		
Location, *n* (%)	Cervical region	10 (12.5)
	Axilla	26 (32.5)
	Groin	44 (55.0)
Removed nodes, median (range)		16 (7–48)
Positive nodes, median (range)		2 (1–19)
Tumour size, median (range)		3.1 (0.5–7.0)
Extranodal growth, *n* (%)	No	55 (68.8)
	Yes	25 (31.2)

**Fig. 1:**
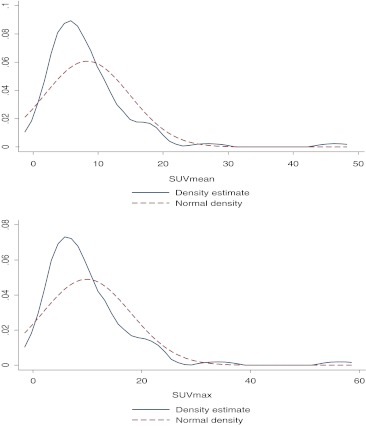
Distributions of the SUVmean and SUVmax values in comparison with the normal (gaussian) distributions

As shown in Fig. 1, the SUV values were not normally distributed (no gaussian distribution). A log-transformation of the SUVmean and SUVmax values provided the best fit to a Gaussian distribution. The log-transformed SUVmean and SUVmax values were not associated with gender (*p* = 0.3 and *p* = 0.4), age (*p* = 0.2 and *p* = 0.1), location of the lymph nodes (*p* = 0.2 for both), number of removed nodes (*p* = 0.4 for both), number of positive nodes (*p* = 0.1 for both) or extranodal growth (*p* = 0.6 for both), respectively. SUV was, however, associated with size of the melanoma metastasis in the lymph node (*p* < 0.001).

### Survival

During the follow-up (median 3.0 years, range: 0.3–8.2 years, in all patients; 5.5 years, 3.1–8.2 years, in patients without an event), 55 patients (68.8 %) developed a recurrence. Overall, 49 patients (61.3 %) died as a result of the recurrent disease and 6 patients with recurrence were still alive at the end of the study. The median SUVmean (used for further analysis) was 6.49 (IQR 4.5–10.9) and the median log(SUVmean) was 1.86.

**Fig. 2:**
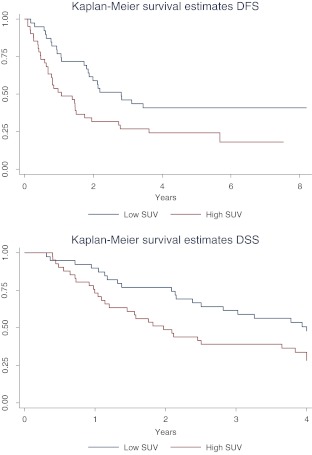
Kaplan Meier curves for DFS and DSS in patients with a high and low SUV

The 5-year DFS in patients with a low SUVmean was 40.9 % (95% CI 25.5–55.7 %) and 24.2 % (95%CI 12.4–38.0 %) in patients with a high SUVmean (*p* = 0.02), as shown in Fig. 2. Webtable 1 shows the univariate and multivariable analysis of DFS, and Table 2 shows the analysis of DFS in relation to SUV. SUVmean was associated with DFS with a hazard ratio (HR) of 1.7 (95% CI 1.0–3.0; *p* = 0.048). Other variables associated with DFS in the multivariable analysis were the number of positive nodes (HR 1.1, 95% CI 1.0–1.2; *p* = 0.02) and the presence of extranodal growth (HR 3.4, 95% CI 1.4–4.2; *p* = 0.003).

**Table 2:** Multivariable analyses of DFS and DSS for the patients with Stage III melanoma

Variable		DFS^a^		DSS^b^	
		Hazard ratio	*p* value	Hazard ratio	*p* value
SUV^c^	Low	1 (reference)	0.048	1 (reference)	0.1
	High	1.74 (1.00–3.00)		1.57 (0.86–2.87)	

^a^Adjusted for positive nodes and extranodal growth (see Webtable).

^b^Adjusted for sex, positive nodes and extranodal growth (see Webtable).

^c^Divided by the median of log(SUVmean) = 1.86.

The 5-year DSS was 47.5 % (95% CI 30.9–62.3) in patients with a low SUVmean and 30.3 % (95% CI 16.7–45.0) for patients with a high SUVmean (*p* = 0.02, Fig. 2). Webtable 2 shows the univariate and multivariable analysis of DSS, and Table 2 shows the analysis of DSS in relation to SUV. SUVmean was not associated with DSS in the multivariable analysis (HR 1.6, 95% CI 0.9–2.9; *p* = 0.1). Female gender was associated with a better DSS in the multivariable analysis (HR 0.5, 95% CI 0.3–0.8; *p* = 0.009).

## Discussion

The present prospective study endorses the prognostic value of SUV in terms of DFS in patients with stage IIIB melanoma who have been optimally staged with FDG PET and CT. The use of SUV as a quantitative parameter of FDG uptake has a long tradition in nuclear medicine [14]. Although its value for predicting tumour response to therapy has become generally accepted, the clinical usefulness and applicability of SUV for prognostic purposes is still under discussion.

As far as the authors are aware there are two other studies (from our group) that addressed the value of SUV in melanoma patients [13, 22]. The first study was a small retrospective study that showed no significant association between SUVmean and survival (*p* = 0.11); however, DFS was significantly decreased in patients with a high SUVmean (*p* = 0.03) [13]. The study by Kruijff et al. showed no association between SUV and survival [22]. There are, however, significant differences between the patients in the two databases, especially in location, number of positive nodes (more in the present study) and percentage of patients with extranodal growth (lower percentage in the present study). Furthermore, the cut-off values in both studies were data-driven and differ. In the present prospective study we confirmed the association found in the retrospective study [13]. There was a significantly decreased DFS in patients with a high SUVmean in the lymph node metastasis (*p* = 0.048). An association between SUVmean and survival was not found. DSS was, however, associated with gender, as reported previously by De Vries et al. [23]. Among 10,538 melanoma patients, women had a superior survival than men even after adjustment for multiple confounding variables [23]. Probably factors other than stage at diagnosis and location reduces mortality risk in female melanoma patients. The associations between extranodal growth and the number of nodes have been reported before: extranodal growth showed a stronger association with DFS than SUVmean [24].

 Some of the primary melanoma characteristics such as Breslow thickness and ulceration were not associated with survival in these patients with macrometastatic disease. Interestingly, a recent study by Balch et al. showed similar results: in patients with nodal micrometastases, multiple covariates independently predicted survival, including several primary melanoma features (thickness, mitotic rate, ulceration, and anatomic site of the primary tumour) [25]. In contrast, in patients with nodal macrometastases, primary melanoma characteristics did not predict survival. Remarkably, there was no clear significant association between tumour size and survival in the present study. A longer follow-up of the patients might have altered these outcomes. Categorization of tumour size into <3 cm or ≥3 cm did not lead to different results (DFS HR 1.7, 95% CI 1.0–3.0), *p* = 0.1; DSS HR 1.7, 95% CI 1.0–3.2, *p* = 0.1). Unfortunately, we were not able to stratify according to tumour size. Further, larger studies should possibly involve stratification according to smaller and larger tumour in the lymph nodes.

We present a single-centre study with standardized PET procedures to obtain homogeneity at that level. Since the measured SUV is a function of many technical and biological factors, the absolute values are only generalizable if these are taken into account. A recent study by Westerterp et al. showed differences in SUV quantification between institutes with different PET scanners [26]. Consequently, standardization of acquisition, reconstruction and data analysis is needed for multicentre trials. When the present study was initiated, there were no guidelines on how to harmonize the results of multicentre studies using different scanners, acquisition and reconstruction protocols. However, provided that the 2010 PET guidelines for trials and clinical practice are followed [27], this is no longer an obstacle, and hopefully the evidence for the potential of quantitative PET in oncology will mature rapidly. In the present study we used the median value of the log-transformed SUVmean for risk stratification. However, there is always change for bias if data-driven cut-off values are used. To be a practical prognostic factor in routine practice, standardization of protocols and cut-off values for SUV should be agreed upon or the methodology to determine the optimal threshold for each centre should be established [17].

As a FDG PET scan as a staging procedure prior to treatment of metastatic melanoma in regional lymph nodes is now performed, determination of SUV values would provide additional information at very little extra cost. In conclusion, in patients with clinical stage III melanoma, a high SUVmean was associated with decreased DFS. If confirmed in larger studies, SUVmean could potentially be used, in addition to the number of positive nodes, tumour size and extranodal growth, as a factor in deciding on adjuvant systemic treatment.

## Electronic supplementary material

Below is the link to the electronic supplementary material.

Supplementary webtable 1 (DOC 40 kb)

Supplementary webtable 2 (DOC 40 kb)
