# Supplementary material for: Prognostic value of the standardized uptake value for 18F-fluorodeoxyglucose in patients with stage IIIB melanoma
Source: Eur J Nucl Med Mol Imaging. 2012 Jul 17;39(10):1592–8. doi: 10.1007/s00259-012-2182-0 (PMC3458194; doi:10.1007/s00259-012-2182-0)
Supplement: Supplementary file 1 — (DOC 40 kb) [file 259_2012_2182_MOESM1_ESM.doc]

Supplementary webtable 1: Univariate and multivariable analysis of the Disease Free Survival

| **Variable** | | **Univariate analysis**  **HR (95%CI) p-value** | | **Multivariable analysis**  **HR (95%CI) p-value** | |
| --- | --- | --- | --- | --- | --- |
| **Gender** | Male  Female | 1 (ref)  0.63 (0.37-1.07) | 0.1 |  |  |
| **Age** | Continuous | 0.99 (0.98-1.01) | 0.8 |  |  |
| **Breslow thickness** | ≤1.0  1.0-2.0  ≥2.0  Unknown primary | 1 (ref)  0.91 (0.38-2.17)  1.05 (0.46-2.39)  0.69 (0.08-5.62) | 0.9 |  |  |
| **Ulceration primary** | No  Yes | 1 (ref)  1.51 (0.81-2.83) | 0.2 |  |  |
| **Nodes removed** | Continuous | 0.98 (0.94-1.02) | 0.3 |  |  |
| **Nodes positive** | Continuous | **1.11 (1.04-1.20)** | **0.003** | **1.09 (1.01-1.18)** | **0.02** |
| **Localization** | Cervical  Axilla  Groin | 1 (ref)  4.17 (1.23-14.1)  3.96 (1.21-12.96) | 0.06 |  |  |
| **Extranodal growth** | No  Yes | **1 (ref)**  **2.85 (1.66-4.90)** | **<0.001** | **1 (ref)**  **2.37 (1.35-4.15)** | **0.003** |
| **Tumor size lymph node** | Continuous | 1.13 (0.98-1.29) | 0.1 |  |  |
| **SUV*** | Low  High | **1 (ref)**  **1.93 (1.13-3.30)** | **0.02** | **1 (ref)**  **1.74 (1.00-3.00)** | **0.048** |

Ref = reference, HR = Hazard Ratio. All significant variables in univariate analysis were entered into the multivariable analysis.
