# Supplementary material for: Prognostic value of the standardized uptake value for 18F-fluorodeoxyglucose in patients with stage IIIB melanoma
Source: Eur J Nucl Med Mol Imaging. 2012 Jul 17;39(10):1592–8. doi: 10.1007/s00259-012-2182-0 (PMC3458194; doi:10.1007/s00259-012-2182-0)
Supplement: Supplementary file 2 — (DOC 40 kb) [file 259_2012_2182_MOESM2_ESM.doc]

Supplementary webtable 2: Univariate and multivariable analysis of the Disease Specific Survival

| **Variable** | | **Univariate analysis**  **HR (95%CI) p-value** | | **Multivariable analysis**  **HR (95%CI) p-value** | |
| --- | --- | --- | --- | --- | --- |
| **Gender** | Male  Female | **1 (ref)**  **0.50 (0.28-0.89)** | **0.02** | **1 (ref)**  **0.46 (0.26-0.82)** | **0.009** |
| **Age** | Continuous | 1.00 (0.99-1.02) | 0.7 |  |  |
| **Breslow thickness** | ≤1.0  1.0-2.0  ≥2.0  Unknown primary | 1 (ref)  0.98 (0.36-2.66)  1.02 (0.39-2.65)  0.85 (0.10-7.24) | 0.9 |  |  |
| **Ulceration primary** | No  Yes | 1 (ref)  1.77 (0.93-3.35) | 0.08 |  |  |
| **Nodes removed** | Continuous | 0.99 (0.95-1.03) | 0.6 |  |  |
| **Nodes positive** | Continuous | **1.10 (1.02-1.18)** | **0.01** | 1.08 (0.99-1.16) | 0.06 |
| **Localization** | Cervical  Axilla  Groin | 1 (ref)  5.94 (1.36-25.76)  4.49 (1.07-18.87) | 0.06 |  |  |
| **Extranodal growth** | No  Yes | **1 (ref)**  **2.00 (1.13-3.54)** | **0.02** | 1 (ref)  1.63 (0.87-3.07) | 0.1 |
| **Tumor size lymph node** | Continuous | 1.74 (0.96-3.15) | 0.07 |  |  |
| **SUV*** | Low  High | **1 (ref)**  **1.82 (1.03-3.22)** | **0.04** | 1 (ref)  1.57 (0.86-2.87) | 0.1 |

Ref = reference, HR = Hazard Ratio. All significant variables in univariate analysis were entered into the multivariable analysis.
